# Supplementary material for: Co-Carbonization of Discard Coal with Waste Polyethylene Terephthalate towards the Preparation of Metallurgical Coke
Source: Materials (Basel). 2023 Mar 30;16(7):2782. doi: 10.3390/ma16072782 (PMC10095968; doi:10.3390/ma16072782)
Supplement: Supplementary file 1 [file materials-16-02782-s001.zip › materials-2269675-supplementary.pdf]

Electronic supporting information

Co-carbonization of discard coal with waste polyethylene terephthalate towards the preparation of metallurgical coke

Sonwabo E. Bambalaza[1*], Buhle S. Xakalashe[1], Yolindi Coetsee[2], Pieter G. van Zyl[2], Xoliswa L. Dyosiba[3], Nicholas M. Musyoka[3], Joalet D. Steenkamp[4]

[1]Pyrometallurgy division, Mintek, 200 Malibongwe drive, Praegville, Johannesburg, 2125, South Africa.

[2]Chemical Resource Beneficiation, North West University, Potchefstroom, 2531, South Africa.

[3]HySA Infrastructure Centre of Competence, Centre for Nanostructures and Advanced Materials (CeNAM), Chemicals Cluster, Council for Scientific and Industrial Research (CSIR), Pretoria 0001, South Africa.

[4]Expert Process Solutions – Glencore, Pyrometallurgy and Furnace Integrity, 6 Edison road, Falconbridge, ON P0M 1S0, Canada.

[*]Corresponding author: sonwabob@mintek.co.za (S.E. Bambalaza)

The following supporting information is a compilation of supporting data towards the synthesis of carbon derived from waste PET (w-PET) using discard coal as the substrate for carbon deposition.

Table S1: Summary of the composition of mixtures used for direct carbonization and co-carbonization processes.

	Mass (g)		
**Method 1**	**w-PET**	**d-coal ash**	**d-coal**
**Direct carbonization**	20	0	0
**Method 2**	**Mass (g)**		
**Co-carbonization**	w-PET	d-coal ash	d-coal#
Composite 1	10	10	0
Composite 2	13.3	6.7	0
Composite 3	15	5	0
Composite 4	16	4	0
Composite 5	12	8	0
Composite 6*	15	0	5

#d-coal = ROM, SM, or BCF

*d-coal used in place of d-coal ash for each of the ROM, SM, and BCF discard coals.

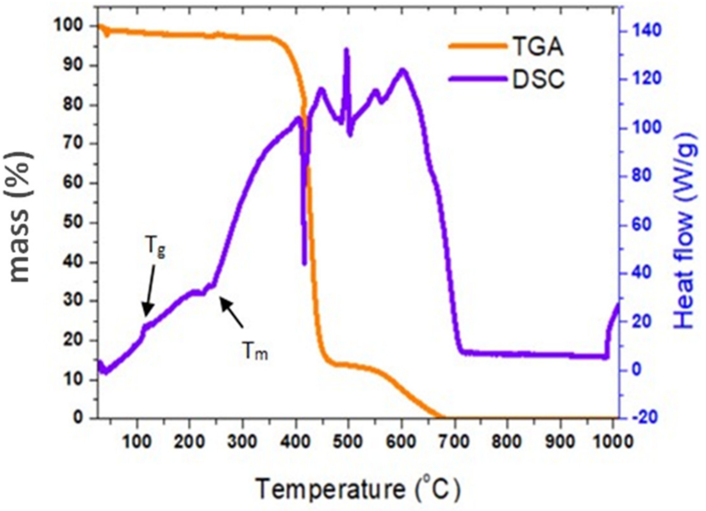

Figure S1: TGA and DSC analysis of w-PET in air at a heating rate of 10°C/min and up to a target temperature of 1000°C.

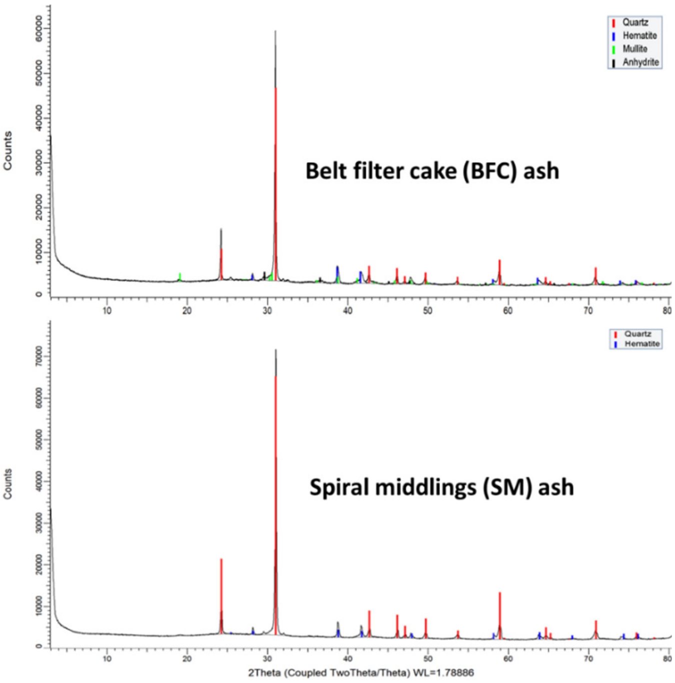

Figure S2: QXRD patterns obtained for ash samples derived from BFC and SM samples. The measured patterns are overlaid with PDF reference diffraction patterns for quartz, hematite, mullite, and anhydride, indicating each chemical's presence or absence in the sample.

Table S2: Summary of the bulk mineralogy obtained for BFC ash and SM ash. The composition of each coal ash type shows relative amounts of compounds in each sample.

Color	Compound identified	Formula	BFC ash	SM ash
□	Quartz	SiO_2	58.6	81.4
□	Hematite	Fe_2O_3	19.1	18.6
□	Mullite	$3Al_2O_3{\cdot}2SiO_2$	13.9	-
□	Anhydrite	$CaSO_4$	8.4	-
**Total**			100.0	100.0

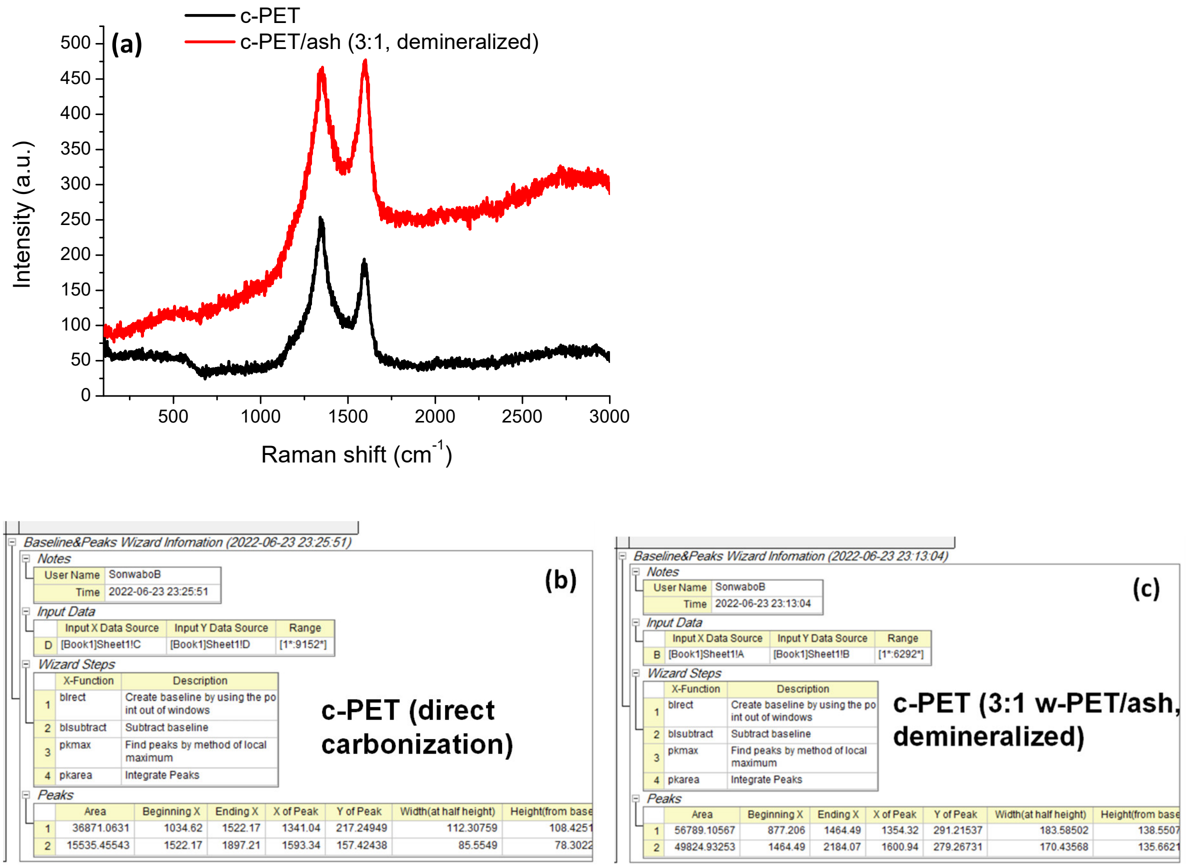

(b) Baseline&Peaks Wizard Infomation (2022-06-23 23:25:51)

Notes

User Name	SonwaboB
Time	2022-06-23 23:25:51

Input Data

	Input X Data Source	Input Y Data Source	Range
D	[Book1]Sheet1!C	[Book1]Sheet1!D	[1*:9152*]

Wizard Steps

	X-Function	Description
1	blrect	Create baseline by using the po int out of windows
2	blsubtract	Subtract baseline
3	pkmax	Find peaks by method of local maximum
4	pkarea	Integrate Peaks

Peaks

	Area	Beginning X	Ending X	X of Peak	Y of Peak	Width(at half height)	Height(from base
1	36871.0631	1034.62	1522.17	1341.04	217.24949	112.30759	108.4251
2	15535.45543	1522.17	1897.21	1593.34	157.42438	85.5549	78.3022

(c) Baseline&Peaks Wizard Infomation (2022-06-23 23:13:04)

Notes

User Name	SonwaboB
Time	2022-06-23 23:13:04

Input Data

	Input X Data Source	Input Y Data Source	Range
B	[Book1]Sheet1!A	[Book1]Sheet1!B	[1*:6292*]

Wizard Steps

	X-Function	Description
1	blrect	Create baseline by using the po int out of windows
2	blsubtract	Subtract baseline
3	pkmax	Find peaks by method of local maximum
4	pkarea	Integrate Peaks

Peaks

	Area	Beginning X	Ending X	X of Peak	Y of Peak	Width(at half height)	Height(from base
1	56789.10567	877.206	1464.49	1354.32	291.21537	183.58502	138.5507
2	49824.93253	1464.49	2184.07	1600.94	279.26731	170.43568	135.6621

Figure S3: Raman spectra of c-PET products obtained by direct carbonization of w-PET and co-carbonization of a 3:1 w-PET/ash composite: (a) Overlayed spectra plotted from raw data (without baseline corrections); (b) – (c) Output results obtained from the integration of the Raman spectra. The tables give calculated values after

baseline corrections have been made which were used to give an estimate of the I_D/I_G ratio.

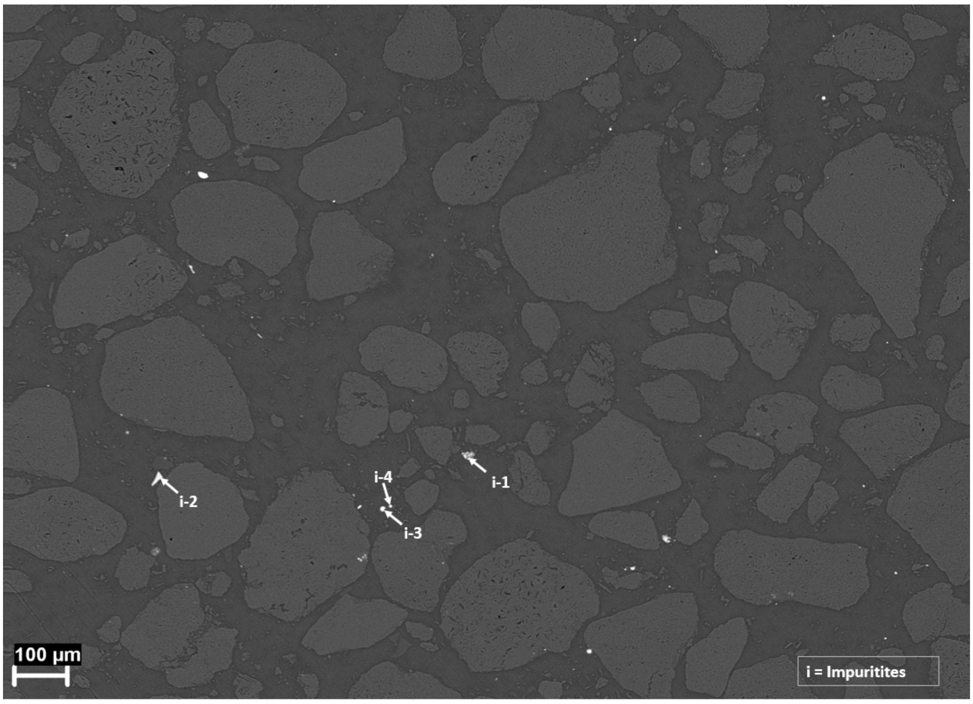

Figure S4: SEM image of the microstructure of c-PET showing marked impurities as identified by spot analysis using EDS spectroscopy (identified phases in Table S2).

Table S3: Quantification of the possible metal oxide phases present in c-PET sample under EDS analysis.

Diagram Ref	Possible Phase	O	Na	Al	Si	K	Ca	Ti	Fe
Impurity – 1	Aluminosilicate	48.6	0.7	17.8	25.8	1.5	3.4	2.2	-
Impurity – 2	Silica	53.3	-	-	46.7	-	-	-	-
Impurity – 3	Ca Aluminosilicate	47.7	-	14.8	26.6	0.6	9.0	0.5	0.7
Impurity – 4	Ti Aluminosilicate	48.1	-	22.0	20.3	1.1	0.9	6.8	0.8

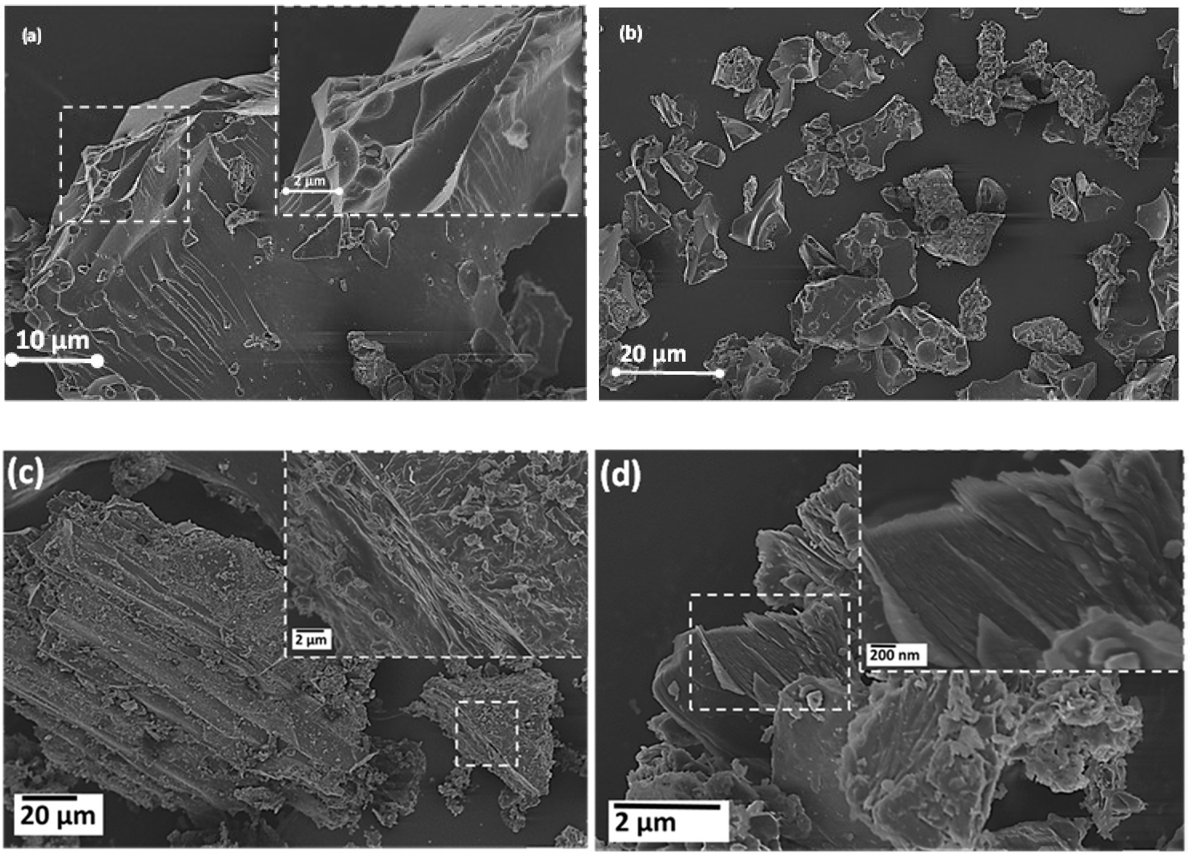

Figure S5: FIB-SEM images showing the morphology of carbon particles obtained for c-PET/d-coal ash composites and commercial metallurgical coke: (a)-(b) Carbon particles derived from demineralized c-PET/d-coal ash composites (insert at 10 000 X magnification), and (c)-(d) Metallurgical coke particles at 5000 X magnification (inserts at 10 000 X magnification)

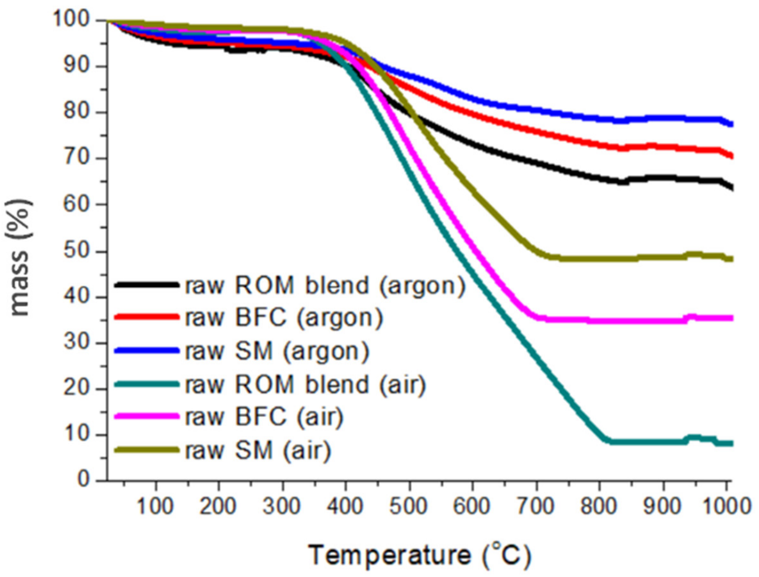

Figure S6: TGA thermograms for raw d-coal samples performed under argon flow (3L/min) and stagnant (ambient air) conditions.

The following equations were used to calculate the fixed carbon content and additional carbon (c-PET) in c-PET/d-coal products:

$FC = 100 - [Ash] - [Volatiles]$ …………………………………………………..(S1)

[Ash] – Ash content of coal (%)

[Volatiles] – Volatile matter content of coal (%)

FC – Fixed carbon content (%)

$[c - PET] = FC_{composite} - FC_{d-coal}$………………………………………………….(S2)

[c-PET] – Additional carbon derived from w-PET (wt%)

$FC_{composite}$ – Fixed carbon content of c-PET/d-coal composite (wt%)

FC_{d-coal} – Fixed carbon content of carbonized d-coal (wt%)

Table S4: Summary of the proximate analysis results obtained for raw discard coal samples. The analysis was done using both TGA and ASTM standard methods.

**Sample**	**Ash content (%)**		**Volatiles, including moisture (%)**		**Fixed Carbon (%)**	
	**TGA**	**ASTM D3174** [1]	**TGA**#	**ASTM D3175 / D3173** [2], [3]	**TGA**	**ASTM D3172** [4]
**Raw ROM blend**	8	7	36	30	56	63
**Raw BFC**	36	34	27	20	37	46
**Raw SM**	48	52	19	16	33	32

In Table S4, the additional PET-derived carbon (c-PET) is calculated as the difference between the fixed carbon contents of carbonized discard coals and their carbonized c-PET/d-coal composite counterparts.

Table S5: Proximate analysis of raw coal samples in comparison to their c-PET/coal composite counterparts. The results are based on TGA and ASTM testing data.

**Sample**	**Ash content (%)**		**Volatiles, including moisture (%)**		**Fixed Carbon (%)**		**Additional fixed PET-derived carbon (%)**	
	**TGA**	**ASTM D3174** [1]	**TGA#**	**ASTM D3175 / D3173** [2], [3]	**TGA**	**ASTM D3172** [4]	**TGA**	**ASTM D3172** [4]
**c-ROM blend**	10	10	15	12	75	78	10	8
**c-PET/c-ROM blend**	7	7	8	7	85	86		
**c-BFC**	40	43	15	15	45	42	16	20
**c-PET/c-BFC**	30	32	9	6	61	62		
**c-SM**	78	72	8	10	14	18	35	34
**c-PET/c-SM**	42	42	9	6	49	52		

#Weight change (%) obtained from the TGA thermogram under an argon flow of 3L/min at 900-950°C.

Table S6: Summary of experimental compressive strength values obtained for commercial metallurgical coke in comparison to the best performing PET/coal composite.

Run	Maximum load at failure (N)	
	Metallurgical coke (commercial)	c-PET/c-ROM blend composite (3:1)
1	3618	25
2	7004	47
3	3677	137
4	3633	44
5	3633	88
6	1334	30
**Average**	**3817**	**62**
**Std deviation**	**1814**	**43**

References

[1] ASTM-D3174, *Standard Test Method for Ash in the Analysis Sample of Coal and Coke from Coal*. West Conshohocken, PA, 2003: ASTM International, 2012.

[2] ASTM-D3173, *Standard Test Method for Moisture in the Analysis Sample of Coal and Coke*. West Conshohocken, PA, 2003: ASTM International, 2011.

[3] ASTM-D3175, *Standard Test Method for Volatile Matter in the Analysis Sample of Coal and Coke*. West Conshohocken, PA, 2003: ASTM International, 2011.

[4] ASTM-D3172, *Standard Practice for Proximate Analysis of Coal and Coke*. West Conshohocken, PA, 2003: ASTM International, 2013.
